# Health Risk Assessment Based on Exposure to Chemicals in Air

**DOI:** 10.3390/ijerph192315813

**Published:** 2022-11-28

**Authors:** Hironari Sakamoto, Shigehisa Uchiyama, Ayana Sato, Tomohiko Isobe, Naoki Kunugita, Hironao Ogura, Shoji F. Nakayama

**Affiliations:** 1 Faculty and Graduate School of Engineering, Chiba University, Chiba 263-8522, Japan; 2 Japan Environment and Children’s Study Programme Office, National Institute for Environmental Studies, Tsukuba 305-8506, Japan; 3 School of Health Sciences, University of Occupational and Environmental Health, Fukuoka 807-8555, Japan

**Keywords:** personal exposure, indoor air quality, health risk assessment, diffusive sampler

## Abstract

Few studies have investigated personal exposure concentrations of not only some volatile organic compounds but also more types of chemicals including acidic gases and acrolein. We measured the personal exposure concentrations of 35 chemicals including these chemicals in indoor and outdoor air in Chiba-shi, Japan, for 7 days in summer and winter to assess the associated health risks in 22 people. The personal exposure concentrations of nitrogen dioxide were higher in winter than in summer, and those of formaldehyde, *p*-dichlorobenzene, and tetradecane were higher in summer than in winter. The personal exposure concentrations were mostly equal to or lower than the concentrations in indoor air, contrary to the results of a lot of previous studies. The high-risk chemicals based on personal exposure concentrations were identified as acrolein (max. 0.43 μg/m^3^), benzene (max. 3.1 μg/m^3^), and hexane (max. 220 μg/m^3^) in summer, and acrolein (max. 0.31 μg/m^3^), nitrogen dioxide (max. 320 μg/m^3^), benzene (max. 5.2 μg/m^3^), formic acid (max. 70 μg/m^3^), and hexane (max. 290 μg/m^3^) in winter. In addition, we estimated personal exposure concentrations according to the time spent at home and the chemical concentrations in indoor and outdoor air. We found that the estimated concentrations of some participants largely differed from the measured ones indicating that it is difficult to estimate personal exposure concentrations based on only these data.

## 1. Introduction

Various volatile chemicals are used in daily products, such as paints, adhesives, and air fresheners, and emitted into indoor air. The concentrations of these chemicals in indoor air are higher than those in outdoor air because of the closed indoor environment. In addition, many people spend 90% of their time indoors [1,2], which may cause indoor air quality (IAQ) to affect human health much more severely than outdoor air quality.

In Japan, IAQ guidelines have been established by the Ministry of Health, Labor, and Welfare (MHLW) for 13 chemicals (formaldehyde, toluene, xylene, *p*-dichlorobenzene, ethylbenzene, styrene, chlorpyrifos, di-*n*-butyl phthalate, tetradecane, di-(2-ethylhexyl)phthalate, diazinon, acetaldehyde, and fenobucarb) [3,4]. However, these target chemicals may be reviewed and updated to reflect the latest scientific findings. Moreover, the types and concentrations of the chemicals in indoor air have been changing in line with change of building materials and consumer products [5]. Therefore, a more comprehensive evaluation of the health risks of pollutants in indoor air is required to include chemicals other than those stated in the MHLW guidelines.

Although IAQ has been widely investigated in Japan [6,7,8,9,10,11], few studies have been conducted to assess personal exposure concentrations to accurately evaluate the health risks of exposure to volatile organic compounds (VOCs). It is important to measure personal exposure because outdoor and indoor monitoring may not reflect individual exposure, and different family members have different life patterns, e.g., time spent at home. Personal exposure concentrations and inhalation health risks have been studied in other countries. Sexton et al. compared the measured personal exposure concentrations with the benchmark concentrations, which were associated with an excess 70-year lifetime carcinogenic risk of 1/100,000 [12]. Payne-Sturges et al. and Sax et al. estimated the cancer risk by multiplying the measured personal exposure concentration by its unit risk, which is described as the probability of developing cancer per 1 µg/m^3^ of inhaled air in 70 years, and then summed these cancer risks for all the measured carcinogenic VOCs [13,14]. Payne-Sturges et al. evaluated noncancer risks by comparing the measured personal exposure concentrations with the inhalation reference concentrations (RfCs) to present a noticeable risk of noncancer effects during a lifetime. Although individual exposure concentrations have been used in few cases in Japan, Wulan et al. measured eight VOCs and compared them with an intake equivalent to an excess cancer incidence rate of 1 × 10^−5^. However, these studies have remained limited to the health risks of only some VOCs. The determination of the overall health risk requires incorporating more types of chemicals, such as acidic gases and acrolein.

Azuma et al. assessed the cancer and noncancer risks of 49 chemicals, including acidic gases and acrolein, based on the measured indoor air concentrations [15]. They determined the RfCs of various chemicals based on the health risks reported by international and national agencies, such as World Health Organization (WHO) and MHLW. These RfCs are useful to evaluate inhalation health risks for a wide range of gaseous chemicals.

In this study, we aimed to reveal the personal exposure concentrations of numerous chemicals, including acidic gases and acrolein, and clarify high-risk chemicals based on the measured personal exposure concentrations using the RfCs determined by Azuma et al. [15]. Thus, we measured the personal exposure, indoor air, and outdoor air concentrations of 35 chemicals, including acidic gases and acrolein, in Chiba-shi, Japan. Chiba-shi is located roughly in the center of Chiba Prefecture and approximately 40 km east of Tokyo, which has a population of 978,000 in an area of 272 km^2^. It was selected as the study area for ease of recruitment and to obtain the representative data in the Greater Tokyo Area.

Additionally, we estimated the personal exposure concentrations according to the indoor and outdoor air concentrations of the chemicals and the time spent at home and compared them with the measured personal exposure concentrations. We also measured the personal exposure concentrations of the family members living in the same house and compared their health risks.

## 2. Materials and Methods

### 2.1. Sampling and Analysis

We studied 22 VOCs (hexane, ethyl acetate, benzene, toluene, butyl acetate, ethylbenzene, *m,p*-xylene, *o*-xylene, *α*-pinene, decane, 2-ethyl-1-hexanol, *d*-limonene, undecane, 1,2,3-trimethylbenzene, *p*-dichlorobenzene, dodecane, tridecane, tetradecane, pentadecane, 2,2,4-trimethyl-1,3-pentanediol monoisobutyrate, hexadecane, 2,2,4-trimethyl-1,3-pentanediol diisobutyrate), 7 carbonyl compounds (formaldehyde, acetaldehyde, acetone, acrolein, 2-butanone, 2-nonenal, nonanal), 5 acidic gases (nitrogen dioxide, sulfur dioxide, hydrogen chloride, formic acid, and acetic acid), and ozone. We used three kinds of diffusive sampling devices (DSDs)—namely, DSD-CX packed with Carboxen 572 particles for measuring VOCs, DSD-BPE/DNPH [16,17] packed with 2,4-dinitrophenyl hydrazine (DNPH) and *trans*-1,2-bis(2-pyridyl) ethylene (BPE) coated silica for measuring ozone and carbonyl compounds, and DSD-TEA [10] packed with triethanolamine (TEA) for measuring acidic gases. These devices had the same structure except for the absorbents. The diffusion filter was made of porous sintered polyethylene with a surface area of 3.93 cm^2^ and a thickness of 1 mm, respectively.

Outlines of the houses and residents in this study are shown in Table 1. In Japan, the temperature difference between summer (July–September) and winter (December–February) is very large, and the concentrations of chemical compounds are expected to change. Therefore, we performed the continuous 7-day sampling for each subject in two seasons: summer (July 2019 to September 2019) and winter (December 2019 to February 2020). In this study, we recruited students of Chiba University to facilitate the investigation and target subjects with similar socioeconomic conditions. A total of 22 participants (A–V) participated. A total of 21 and 20 people were sampled in the summer and the winter, respectively, of which 19 people were sampled in both seasons. For personal exposure, indoor air, and outdoor air measurements, samplers were placed near the participants’ breasts, near the center of the living room, and at the veranda of the participants’ house, respectively, in the same manner of the previous studies [18,19,20]. We asked the participants to keep a record of their time spent at home during the sampling period.

Additionally, we also studied the personal exposure concentrations of the family members living in the same house to compare their risk of exposure to the chemicals.

Personal exposure measurements were totally performed on 61 adults (20 years of age and older) including family members, and all participants provided informed consent. For the sampling and analysis of each device, we followed the procedure described in our previous study and used the same analyzing apparatus [21].

### 2.2. Risk Characterization

We evaluated health risks of each chemical based on the hazard quotient (HQ), where a higher HQ indicates a higher health risk, which was calculated using the reference concentration determined by Azuma et al. [15], as follows:

HQ = *C_P_*/RfC (1)

where RfC is the inhalation reference concentration, and *C_P_* is the measured personal exposure concentration. For noncancer effects, Azuma et al. determined the RfC for each chemical according to the World Health Organization air quality guidelines, the estimated no observed adverse effect leRDvel, or the lowest observed adverse effect level reported by international and national agencies, such as WHO and MHLW. For cancer effects, Azuma et al. defined RfCs as concentrations associated with an excess lifetime carcinogenic risk of 1/100,000. We used the same RfCs determined by Azuma et al. to calculate HQ values using Equation (1).

### 2.3. Estimation of Personal Exposure Concentration

The personal exposure concentration can be considered as the time-weighted average of the concentration in each microenvironment where the individual stays [1,18,22]. Since only indoor and outdoor air concentrations were measured in this study, we estimated the personal exposure concentration for each participant using the following equation:

*C_P_* = (*IH*·*C_I_* + *OH*·*C_O_*)/(*IH* + *OH*) (2)

where *C_P_* is the personal exposure concentration (μg/m^3^), *IH* is the time spent at home (min), *OH* is the time spent outside the home (min), and *C_I_* and *C_O_* are the indoor and outdoor air concentrations, respectively. We examined the capability of this simple model to estimate personal exposure concentrations because it is easier to measure indoor and outdoor air concentrations than to measure personal exposure concentrations, for which the participants should wear the sampler all day.

### 2.4. Statistical Analysis

Statistical analyses were performed using nonparametric methods because the concentration distributions for many chemicals were skewed. To compare the results in summer and winter, the Wilcoxon signed-rank test was used. To compare personal exposure, indoor air, and outdoor air concentrations, the Bonferroni correction was applied to the test for multiple comparisons. We calculated Spearman correlation coefficients to assess the relationship between the measured and estimated concentrations. The significance level was set to 5% (*p* < 0.05) in all tests. All statistical analyses were performed using R software, version 4.0.2. [23], and some graphs were drawn using the ggplot2 package [24].

## 3. Results

Table 2 shows the personal exposure, indoor air, and outdoor air concentrations of the selected chemicals which were identified as high-risk chemicals in this study, targeted in the the MHLW guidelines, or ozone. The Wilcoxon signed-rank test was performed to compare the concentrations in winter and summer for the subjects who participated in both samplings (*n* = 19). Significant differences were noted in personal exposure concentrations between summer and winter for formaldehyde, *p*-dichlorobenzene, tetradecane, ozone, and nitrogen dioxide (Table 2). For these chemicals except ozone, significant differences in indoor concentrations between the seasons were also detected. Regarding these chemicals, the personal exposure and indoor concentrations were higher in winter than in summer for nitrogen dioxide, whereas they were higher in summer than in winter for the other chemicals.

**Figure 1:**
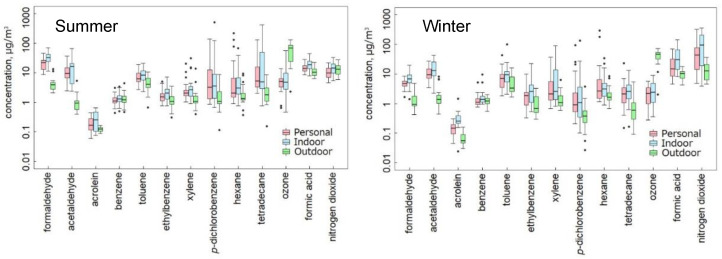
Boxplots of the personal, indoor, and outdoor concentrations of the selected chemicals. The (**left**) and (**right**) panels show the data in summer and winter, respectively. Boxplots represent the median and 25th and 75th percentiles. Whiskers represent minimum and maximum within 1.5 IQR (Interquartile Range, i.e., the range between the 25th and 75th percentile). Dots represent outliers.

Figure 1 shows the boxplots of the personal exposure, indoor air, and outdoor air concentrations of the chemicals listed in Table 2. The Wilcoxon signed-rank test with Bonferroni correction was performed to compare these matched concentrations measured at three different locations. In summer, significant differences were observed among them except for benzene and nitrogen dioxide. The indoor air concentrations were significantly lower than the outdoor air concentrations for ozone but significantly higher for the rest of the chemicals. Furthermore, the personal exposure concentrations were significantly lower than the indoor air concentrations for formaldehyde, ethylbenzene, and formic acid. In winter, for all the chemicals listed in Table 2, significant differences were obtained among the personal exposure, indoor air, and outdoor air concentrations. While the differences between the indoor and outdoor air concentrations were not significant for benzene, the indoor air concentrations were significantly higher than the outdoor air concentrations for the other chemicals except ozone. Moreover, the personal exposure concentrations were significantly lower than the indoor air concentrations for formaldehyde, acetaldehyde, benzene, toluene, ethylbenzene, xylene, formic acid, and nitrogen dioxide.

**Figure 2:**
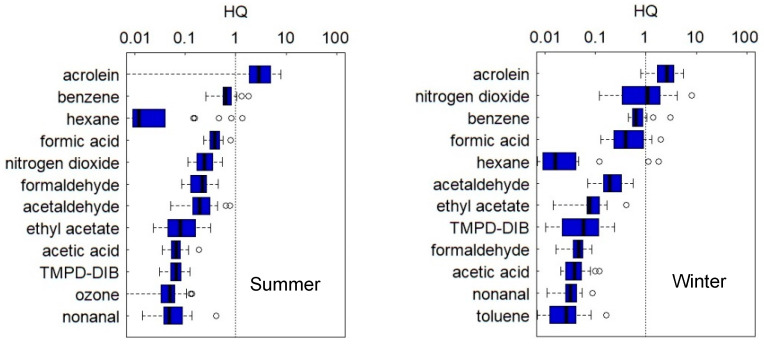
Boxplots of hazard quotients for the personal exposure concentrations. The (**left**) and (**right**) panels show the data in summer and winter, respectively.

Figure 2 shows the boxplots of the HQ calculated from the personal exposure concentrations in summer and winter. In this figure, the chemicals are arranged in the descending order of the percentage of HQ ≥ 1, and if the percentage of HQ ≥ 1 is equal to zero, they are arranged in the order of the median of HQ from high to low. In this study, the chemicals with an HQ higher than 1 were defined as high-risk ones. These chemicals (with the percentage of HQ ≥ 1 shown in parentheses) were acrolein (95%), benzene (14%), and hexane (4.8%) in summer, and acrolein (95%), nitrogen dioxide (55%), benzene (20%), formic acid (20%), and hexane (10%) in winter.

**Figure 3:**
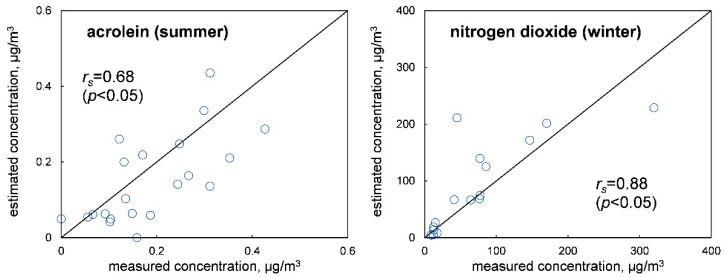
Comparison of the measured concentrations with the estimated concentrations of acrolein in summer (the **left** panel) and nitrogen dioxide in winter (the **right** panel).

The personal exposure concentrations of the high-risk chemicals were estimated according to Equation (2) with the measured indoor and outdoor air concentrations and the time spent at home. Figure 3 shows the comparison of the measured personal exposure concentrations with the estimated ones for acrolein in summer and nitrogen dioxide in winter. For all the high-risk chemicals, the Spearman correlation coefficients (*r_s_*) between the measured and estimated concentrations were more than 0.5 (*p* < 0.05), which indicated monotonically increasing relationships. However, the estimated personal exposure concentrations of some participants markedly differed from the measured ones, as shown in Figure 3. The maximum or minimum differences between the estimated and measured concentrations (with the ratios of the values to the RfC shown in parentheses) were as follows: in summer, acrolein, −0.17 μg/m^3^ (−3.2); benzene, +0.89 μg/m^3^ (+0.52); hexane, −180 μg/m^3^ (−1.1); and in winter, acrolein, +0.80 μg/m^3^ (+15); nitrogen dioxide, +170 μg/m^3^ (+4.1); benzene, +1.1 μg/m^3^ (+0.62); formic acid, +71 μg/m^3^ (+2.0); hexane, −290 μg/m^3^ (−1.8).

**Figure 4:**
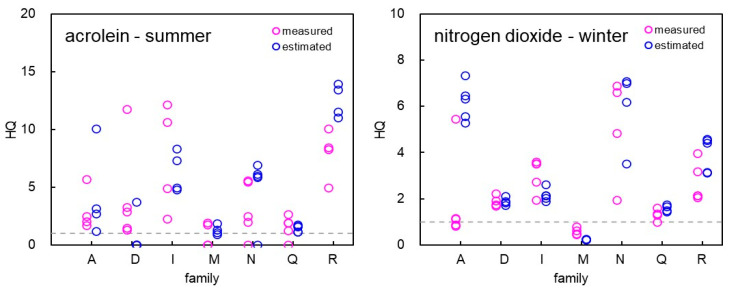
Distributions of hazard quotients for the members of each family. The (**left**) panel shows the data of acrolein in summer, and the (**right**) panel shows the data of nitrogen dioxide in winter. The red and green circles indicate values based on the measured and estimated personal exposure concentrations, respectively.

The results of measuring each personal exposure concentration of one resident in one house are described above. Moreover, we also measured the personal exposure concentrations of the family members in each house. Figure 4 shows the HQ distributions calculated from each family member’s measured concentration for acrolein in summer and nitrogen dioxide in winter (for families consisting of more than two members). Figure 4 also shows the HQ calculated from the estimated concentrations derived using Equation (2). As shown in the figure, there were large differences in HQ values among the residents living in the same house. Regarding high-risk chemicals, the means of the differences between the maximum and minimum HQ in each house were as follows (those calculated using estimated concentrations are shown in parentheses): in summer, acrolein, 3.9 (1.9); benzene, 0.39 (0.04); hexane, 0.19 (0.01); and in winter, acrolein, 5.5 (2.3); nitrogen dioxide, 2.1 (1.2); benzene, 0.58 (0.30); formic acid, 1.0 (0.55); hexane, 0.20 (0.01).

## 4. Discussion

### 4.1. Concentrations of Selected Chemicals

This study revealed the personal exposure, indoor air, and outdoor air concentrations, in Chiba-shi, Japan (Table 2 and Figure 1). Regarding the chemicals listed in Table 2, the personal exposure and indoor concentrations were significantly higher in winter than in summer for nitrogen dioxide, whereas they were significantly higher in summer than in winter for formaldehyde, *p*-dichlorobenzene, and tetradecane. It indicated that the seasonal variations in their indoor concentrations considerably affect their personal exposure concentrations since their indoor concentrations were higher than the outdoor concentrations for these chemicals.

In our previous research, the concentrations of nitrogen dioxide were higher in winter than in summer, especially in houses using kerosene or gas heaters [10,21]. Thus, the results of nitrogen dioxide in this study were possibly influenced by the same emission sources. Although the indoor concentration of formaldehyde was reported to be higher in summer than in winter [25], the indoor concentrations of various types of VOCs including benzene, toluene, ethylbenzene, and xylene (BTEX) were reported to be higher in winter than in summer [26,27,28,29,30,31] mainly because of lower exchange rates [28]. However, Jia et al. reported small or inconsistent seasonal changes [32] and considered that they occurred because air exchange rates were not substantially different between seasons owing to the use of air conditioners in summer. In this study, seasonal concentration differences were insignificant for many VOCs including BTEX because air exchange rates were not considerably different between seasons in Japan.

Significant differences were detected among the matched personal exposure, indoor air, and outdoor air concentrations for most of the chemicals listed in Table 2. The indoor air concentrations were significantly higher than the outdoor air concentrations except for benzene, nitrogen dioxide, and ozone in summer, and except for benzene and ozone in winter. It indicated the presence of emission sources indoors for these chemicals. Furthermore, the personal exposure concentrations were significantly lower than the indoor air concentrations for formaldehyde, ethylbenzene, and formic acid in summer, for formaldehyde, acetaldehyde, benzene, toluene, ethylbenzene, xylene, formic acid, and nitrogen dioxide in winter. In addition, for the other chemicals except tetradecane and ozone in summer, the medians of the personal exposure concentrations were lower than those of the indoor air concentrations, although the differences were not significant.

A study conducted on children aged 7 to 13 showed that the personal exposure concentrations were lower than the indoor air concentrations [33], as we obtained in this study. However, many previous studies reported that the personal exposure concentrations were higher than the indoor air concentrations [12,30,34,35,36]. Ilgen et al. reported that the personal exposure concentrations were higher than the indoor air concentrations for working people partly because of the exposures in workplaces and cars, while they were at the same level as the indoor air concentrations for nonworking people [30]. Delgado-Saborit et al. considered that these higher personal exposure concentrations were caused by personal activities such as photocopying, use of washing materials, and exposure to environmental tobacco smoke, which increased VOC concentrations locally [34]. Thus, the results of this study suggested that the personal exposure concentration might be less influenced by these factors. However, for hexane, some personal exposure concentrations were much higher than the matched indoor air concentrations obtained in this study, probably because of the exposures in workplaces, where hexane was used as a solvent

Table 3 shows the comparison of personal exposure concentrations measured in this study and those obtained in previous studies [12,14,19,20,33,34,37,38] for selected chemicals (the study by Shuai et al. was conducted at the exposed area near a dyeing industrial complex and the controlled area in Korea). The concentrations of formaldehyde and acetaldehyde in this study were comparable to those obtained in the USA. The concentrations of benzene, toluene, and ethylbenzene in this study were at the same level as or slightly lower than those obtained in the USA, Canada, and Europe, whereas they were considerably lower than those measured in Turkey and Korea. The concentration of *p*-dichlorobenzene in this study was slightly higher than those in USA and Canada in summer, whereas it was close to them in winter. In short, the concentrations of the chemicals in this study were comparable to or lower than those in previous studies except *p*-dichlorobenzene in summer.

### 4.2. Risk Characterization Based on HQ

According to the HQ for the personal exposure in this study, the high-risk chemicals were acrolein, benzene, and hexane in summer, and acrolein, nitrogen dioxide, benzene, formic acid, and hexane in winter (Figure 2). The risks of acrolein were especially high in both seasons. In addition, for hexane, the HQ of the most subjects were much lower than 1 as shown in Figure 2.

Azuma et al. evaluated health risks by measuring the indoor air concentrations of 49 chemicals in 602 houses in Japan and found that the high-risk chemicals were acrolein, nitrogen dioxide, benzene, formic acid, and hydrogen chloride [15], which almost agreed with the results of this study.

Although we did not conduct further investigations to find emission sources, it was reported that acrolein is produced by heating while cooking and emitted by wooden materials [39], benzene is produced by burning activities (e.g., gas heating [40], cooking [41], incense burning [42], and smoking [43]), and nitrogen dioxide and formic acid are produced using kerosene or gas heaters [10]. In this study, for nitrogen dioxide, the substantially increased concentrations in winter suggested the influence of these sources. For benzene, the indoor air concentrations could be influenced by the outdoor air concentrations since both concentrations were similar. For hexane, the subjects with HQ ≥ 1 were likely exposed in their workplaces.

### 4.3. Estimation of Personal Exposure Concentration

We estimated the personal exposure concentrations of the highest-risk chemicals according to the measured indoor and outdoor air concentrations and the time spent at home (Figure 3). However, for some participants, there were larger differences than RfCs between the estimated and measured personal exposure concentrations, as shown before. The exposures outside of the residential indoor or outdoor air could explain the large differences. For example, in the case of hexane, the differences were probably caused by the exposures in the workplaces. Another reason for the large concentration differences could be the distributions of the indoor air concentrations in the houses. In our previous study, we found that the indoor air concentrations considerably differed for the rooms of the same house [21]. In such a house, the estimated and measured personal exposure concentrations were possibly different largely if the subject spent long time at home out of the room where the indoor air measurement was carried out. However, in this study, we did not focus on the distributions of the indoor air concentrations in the houses as we studied the indoor air concentration only in the living room in each house. These results show that the health risks could be much different from the real ones if they were evaluated based on the estimated personal exposure concentrations.

### 4.4. Difference in Health Risks between Family Members

We also calculated the HQ of the family members in each house from the measured personal exposure concentrations (Figure 4) to survey the differences in the health risks between these members because the personal exposure concentrations can be remarkably different between the residents living in the same house, as we reported previously [21]. HQ values were largely different among the residents living in the same house, and in some cases, there were subjects with HQ < 1 and HQ ≥ 1 living in the same house. In general, as we show the means of the differences between the maximum and minimum HQ in each house, the higher the health risk of the chemical is, the larger is the difference in the health risks between the residents living in the same house.

Additionally, we calculated the HQ of the family members from the estimated concentrations derived using Equation (2). The differences in HQ derived from the measured concentrations were mostly larger than those derived from the estimated ones because the former ones were influenced by many factors, including the differences in the microenvironments where each participant stayed, whereas the latter depended only on the differences in the time each subject spent at home. Therefore, for accurate health risk assessment, personal exposure concentrations should be measured even if the participants live in the same house.

## 5. Conclusions

By evaluating personal exposure concentrations of various chemicals in Chiba-shi, Japan, we revealed the following conclusions:

First, the personal exposure concentrations for the selected VOCs in this study were almost equivalent to or lower than those reported in previous studies, except for *p*-dichlorobenzene in summer. The personal exposure and indoor air concentrations for nitrogen dioxide were higher in winter than those in summer, whereas those for formaldehyde, *p*-dichlorobenzene, and tetradecane were higher in summer than those in winter. There were no significant differences between the seasons for BTEX, whose indoor air concentrations were reported to be higher in winter than in summer in many previous studies [26,27,28,29,30,31]. In addition, for most of the chemicals, the personal exposure concentrations were the same level as or lower than the indoor air concentrations.

Second, the HQ values calculated from the measured personal exposure concentrations identified the high-risk chemicals as acrolein, benzene, and hexane in summer and acrolein, nitrogen dioxide, benzene, formic acid, and hexane in winter. These chemicals except hexane were in line with the high-risk chemicals found by Azuma et al. [15]. The participants with HQ ≥ 1 for hexane were probably exposed in their workplaces.

Furthermore, there were cases in which the personal exposure concentrations estimated from the indoor and outdoor air concentrations and the time spent at home differed from the measured personal exposure concentrations by more than the RfCs. Additionally, the differences in HQ based on the measured concentrations between the family members in each house were generally larger than those based on the estimated concentrations, suggesting that it is difficult to estimate personal exposure concentrations using only indoor and outdoor air concentrations and that the health risks evaluated using such estimated personal exposure concentrations cannot represent the real ones. Therefore, direct measurements of personal exposure concentrations are necessary.

We found the high-risk chemicals including acrolein by directly measuring the personal exposure concentrations. However, we could recruit only 22 houses in the specific area in Japan. Thus, it is important to further study the personal exposure concentrations of critical chemicals and reveal their health risks since the insights obtained can be utilized in future epidemiological studies.

The personal sampler used in this study is extremely lightweight and unbreakable, and can be worn even by children. Therefore, it is expected to be applied to individual exposure assessment in large-scale epidemiological surveys such as the Japan Environmental and Children’s Study (JECS) [44].

## Figures and Tables

**Table 1:** Outlines of the houses and residents investigated in this study.

Family	House			Resident	
	Type	Structure	Age	Number	Age
A	apartment	RC	30	4	41 (20–62)
B	apartment	RC	30	2	35 (19–51))
C	detached	wooden	20	1	66
D	apartment	RC	27	5	34 (16–53)
E	detached	wooden	10	4	37 (19–55)
F	detached	wooden	33	3	40 (29–51)
G	detached	wooden	16	5	40 (21–53)
H	apartment	RC	50	2	32 (19–45)
I	apartment	RC	26	4	32 (19–50)
J	apartment	RC	20	2	35 (15–53)
K	apartment	RC	33	4	41 (24–57)
L	detached	wooden	48	4	36 (17–53)
M	apartment	RC	15	4	33 (17–49)
*n*	apartment	RC	28	5	22 (5–41)
O	apartment	RC	24	3	34 (19–49)
*p*	apartment	RC	14	2	21 (20–23)
Q	apartment	RC	10	5	26 (49–45)
R	apartment	RC	35	4	35 (18–52)
S	apartment	SRC	31	2	62 (59–62)
T	detached	wooden	4	2	31 (3–48)
U	detached	wooden	43	2	43 (17–81)
V	apartment	SRC	46	5	39 (19–56)

Age of resident is mean (min.–max.) value. Abbreviations: RC: reinforced concrete construction, SRC: steel reinforced concrete construction.

**Table 2:** Summary of personal exposure concentrations (P), indoor air concentrations (I), and outdoor air concentrations (O) for selected chemicals (μg/m^3^).

Compound		Summer				Winter				*p*-Value *
		*n*	Median	Min.	Max.	*n*	Median	Min.	Max.	
formaldehyde	P	21	22	8.7	46	20	4.7	1.7	8.5	<0.05
	I	21	33	11	71	20	6.7	1.4	20	<0.05
	O	21	3.9	2.1	13	19	0.94	0.42	4.9	<0.05
acetaldehyde	P	21	9.3	2.5	37	20	9.3	3.4	27	0.65
	I	21	16	2.4	65	20	13	2.2	43	0.74
	O	21	0.94	0.40	5.3	19	1.4	0.43	8.3	<0.05
acrolein	P	21	0.16	0.00	0.43	20	0.14	0.04	0.31	0.37
	I	21	0.25	0.00	0.64	20	0.24	0.00	1.4	0.89
	O	21	0.00	0.00	0.16	19	0.00	0.00	0.16	0.38
benzene	P	21	1.1	0.43	3.1	20	1.1	0.76	5.2	0.84
	I	21	1.3	0.50	3.4	20	1.4	0.96	9.7	0.11
	O	21	1.2	0.46	4.4	19	1.2	0.55	2.1	0.92
toluene	P	21	6.2	2.7	19	20	6.9	1.9	44	1.0
	I	21	8.2	2.3	21	20	9.2	2.1	99	0.80
	O	21	4.1	0.66	11	19	3.3	1.7	16	0.58
ethylbenzene	P	21	1.5	0.76	5.0	20	1.8	0.32	9.8	0.89
	I	21	2.0	0.77	7.1	20	2.6	0.53	23	0.37
	O	21	1.0	0.30	3.5	19	0.67	0.29	3.3	0.55
xylene	P	21	2.0	1.0	20	20	2.1	0.66	38	0.28
	I	21	2.6	0.90	31	20	2.6	0.80	91	0.42
	O	21	1.1	0.40	14	19	1.1	0.59	6.3	0.83
*p*-dichlorobenzene	P	21	3.2	0.86	140	20	0.89	0.21	95	<0.05
	I	21	3.5	0.66	500	20	1.0	0.10	140	<0.05
	O	21	1.1	0.11	8.8	19	0.37	0.03	3.7	<0.05
hexane	P	21	2.0	0.88	220	20	2.6	1.1	290	0.71
	I	21	3.0	0.76	67	20	3.1	0.89	35	0.39
	O	21	1.3	0.37	9.2	19	1.6	0.67	8.3	0.87
tetradecane	P	21	5.1	2.0	130	20	2.1	0.15	23	<0.05
	I	21	4.8	0.76	430	20	2.5	0.16	13	<0.05
	O	21	1.8	0.16	8.6	19	0.59	0.09	5.4	<0.05
ozone	P	21	5.0	0.65	14	20	2.1	0.28	5.7	<0.05
	I	21	4.7	0.47	55	20	2.4	0.36	9.0	0.10
	O	21	68	2.3	130	19	46	2.0	72	<0.05
formic acid	P	21	14	8.5	29	20	15	4.6	70	0.17
	I	21	18	7.9	45	20	33	8.1	150	<0.05
	O	21	10	6.4	23	19	10	4.3	18	0.07
nitrogen dioxide	P	21	9.7	4.6	22	20	43	4.8	320	<0.05
	I	21	14	5.3	33	20	94	3.9	360	<0.05
	O	21	13	5.9	27	19	12	4.6	36	0.67

* *p*-Value results obtained for the difference between summer and winter data.

**Table 3:** Comparison of the personal exposure concentrations (µg/m^3^) of the selected chemicals in this study and those obtained in previous studies.

Location	Season	Year	FA	AA	BZ	TL	EB	DCB	Note	Reference
Minneapolis/St. Paul, USA	Spring Summer Fall	1999	–	–	3.2	17	2.2	0.4	Median	Sexton et al. (2004) [12]
New York, USA	Winter Summer	1999	17	13	3.3	–	1.9	9.7	Median	Sax et al. (2006) [14]
Los Angeles, USA	Winter Fall	2000	21	11	4.2	–	3.1	5.3		
Minneapolis, USA	Spring	2000	–	–	1.5	7.7	0.9	1.3	Median	Adgate et al. (2004) [33]
	Winter	2000	–	–	2.1	7.7	1.0	1.0		
Valencia, Spain	Spring Winter Fall	2003–2005	–	–	3.1	11	2.9	–	Median	Llop et al. (2010) [37]
Sabadell, Spain	Spring Winter Fall	2005–2006	–	–	1.0	13	1.4	–		
Windsor, Canada	Summer	2005	–	40	2.0	–	3.6	0.78	Geometric mean	Khanchi et al. (2015) [38]
	Winter	2005	–	20	1.7	–	1.8	0.53		
London, etc., UK	Throughout the year	2005–2007	–	–	2.2	20	3.2	–	Mean	Delgado-Saborit et al. (2011) [34]
Kocaeli, Turkey	Summer	2006	–	–	8.3	35	9.2	–	Median	Pekey and Arslanbaş (2008) [19]
	Winter	2006, 2007	–	–	9.3	52	15	–		
Seogu district of Daegu, Korea (Exposed area)	Summer	2013	–	–	10	130	18	–	Mean	Shuai et al. (2018) [20]
	Fall	2013	–	–	3.6	170	2.4	–		
Suseonggu district of Daegu, Korea (Controlled area)	Summer	2013	–	–	6.1	27	10	–		
	Fall	2013	–	–	2.0	89	2.0	–		
Chiba, Japan	Summer	2019	22	9.3	1.1	6.2	1.5	3.2	Median	This study
	Winter	2019, 2020	4.7	9.3	1.1	6.9	1.8	0.89		

Abbreviations: FA: formaldehyde, AA: acetaldehyde, BZ: benzene, TL: toluene, EB: ethylbenzene, DCB: *p*-dichlorobenzene.

## Data Availability

The dataset generated during the current study is not publicly available, but is available from the corresponding author on reasonable request.
